# Characterizing the food and physical activity environments of selected public schools in Region IV-A, Philippines

**DOI:** 10.3389/fpubh.2025.1694267

**Published:** 2025-11-18

**Authors:** Maria Julia Golloso-Gubat, Cherrie Anne D. L. Andres, Alexandra Lyne E. David, Patricia Muriel G. Aquino, Ma. Justine Camille S. Sabino, Jason Paolo H. Labrador

**Affiliations:** Nutrition and Food Research and Development Division, Department of Science and Technology-Food and Nutrition Research Institute, Taguig, Philippines

**Keywords:** school food environment, physical activity environment, adolescent nutrition, food vendors, Philippines, school health and nutrition policy, spatial analysis

## Abstract

**Introduction:**

Schools are key settings for shaping the dietary and physical activity behaviors of adolescents. In the Philippines, limited evidence exists on the characteristics of school nutrition environments, constraining efforts to design effective interventions.

**Methods:**

This study characterized the food and physical activity environments of selected public schools in Region IV-A, Philippines. A descriptive, cross-sectional design was employed in six purposively selected public schools representing both urban and rural settings. Data were collected through a student survey (*n*=178), geospatial mapping of food vendors within a 150-meter radius of each school, and direct observation of school-based physical activity infrastructure. Descriptive statistics and spatial analysis using the Quantum Geographic Information System (QGIS) were applied to assess vendor density and environmental features.

**Results:**

School canteens were identified as consistent food sources, yet students were frequently exposed to and purchased items from external food vendors. Vendor density was highest within 50 meters of school gates, especially in urban areas. Physical activity infrastructure varied across schools. While basketball courts and open spaces were common, spatial constraints in schools limited opportunities for movement.

**Discussion:**

Findings highlight the influence of school environments on adolescents’ access to food and physical opportunities. Strengthening the integration of national nutrition policies with local government regulations, alongside regular monitoring and evaluation, is essential to ensure policy coherence and impact. Moreover, school-based interventions should be complemented by local measures that regulate food access near schools and enhance physical activity infrastructure to better support adolescent health and well-being.

## 1 Introduction and literature review

Adolescence is a critical developmental stage characterized by rapid physical, emotional, psychosocial, and cognitive changes, requiring adequate and balanced nutrition to support healthy growth. Despite this, malnutrition during adolescence remains a pressing global public health concern (1). In the Philippines, the prevalence of overweight and obesity among adolescents has steadily increased in recent years (2). Moreover, a substantial proportion (82.7%) of Filipino adolescents were found to be insufficiently physically active, falling short of the recommended daily physical activity levels (2). These concerning trends have been linked to obesogenic environments that promote the consumption of energy-dense, nutrient-poor foods, and offer limited opportunities for physical activity (3, 4). Given that schools represent a primary setting where these behaviors can be shaped and reinforced, examining the school nutrition environment is deemed essential.

The school nutrition environment encompasses the various elements within and around the school setting that collectively support the students’ optimal nutrition, including the availability of healthy food and beverages, the dissemination of accurate and consistent nutrition messages, access to supportive nutrition services, and opportunities to cultivate healthy eating and physical activity habits (5). This environment plays a crucial role in shaping the health behaviors of children and adolescents, given the substantial amount of time they spend in school and the influence of institutional policies, infrastructure, and social norms. Evidence indicates that healthier school food and physical activity environments are associated with increased student engagement in physical activity, reduced sedentary behavior, and improved weight-related outcomes, including lower body mass index (BMI) (3, 6). Food availability and accessibility, in particular, have been consistently identified as key determinants of adolescents’ dietary behaviors (7), while the presence of school-based physical activity facilities has been positively linked to higher levels of physical activity (8). However, despite this evidence, the prevailing conditions in many Philippine schools reflect environments that may not fully support these health-promoting behaviors.

Several studies in the Philippines have identified characteristics of obesogenic environments that may contribute to unhealthy dietary behaviors among children and adolescents. Recent findings showed that Filipino public junior high school students consume excessive amounts of sodium, free sugars, and sugar-sweetened beverages (9). This observation aligns with earlier studies showing that Filipino schoolchildren and adolescents have nutritionally inadequate diets with low dietary diversity (10, 11). Within school settings, inexpensive processed snacks are often more readily available than healthier local staples, while opportunities for physical activity remain limited despite the availability of facilities. In addition, the implementation of school-based obesity prevention programs appears inconsistent (12, 13). Beyond school settings, children and adolescents in the Philippines are also exposed to aggressive marketing of unhealthy foods (14). The pervasive promotion of these products on social media further reinforces unhealthy eating behaviors by depicting such foods and beverages as enjoyable, family-oriented, or even healthful (15).

To promote healthy eating habits and improve the dietary intake of schoolchildren, the Department of Education (DepEd) in the Philippines has issued several policies governing food provision and sales in schools. The DepEd Order No. 08 s. 2007 or the *Revised Implementing Guidelines on the Operation and Management of School Canteens in Public Schools* mandate that only nutrient-rich foods such as root crops, rice and corn products, fruits, vegetables, and fortified items be sold, while the sale of sugar-sweetened beverages and foods without the *Sangkap Pinoy* Seal is prohibited (16). Complementing this, the DepEd Order No. 13 s. 2017 or the *Policy on Healthy Food and Beverage Choices in Schools* introduced a color-coded system: green (serve every day), yellow (serve one to three times a week), and red (not recommended for sale) (17). While these frameworks demonstrate a strong policy foundation for promoting school nutrition, gaps remain in implementation, monitoring, and evaluation, and the extent of policy integration at the school level has yet to be fully assessed. Furthermore, there is a notable lack of locally generated data on school nutrition environments in the Philippines, limiting the ability of policymakers, educators, and public health practitioners to design evidence-informed, context-specific, and targeted interventions that effectively promote healthy eating and active lifestyles among students.

This study aims to characterize the school nutrition environment in selected public schools in Region IV-A, Philippines. Specifically, it assesses the types and availability of food options, the density and proximity of food sources, and the presence of physical activity facilities within and around the school premises.

## 2 Materials and methods

This study employed a descriptive, cross-sectional design to assess the school food and physical activity environments in selected public schools in Region IV-A, Philippines.

### 2.1 Study sites

Data were collected from six (6) public schools across four provinces in Region IV-A, which was purposively selected for its demographic and nutrition situation relevance. The region has approximately 2.9 million youth, accounting for 14.5% of the country’s total youth population (18), making it one of the most suitable areas for examining school-related health and nutrition concerns. In addition, Region IV-A consists of provinces that demonstrate clear rural–urban gradients, ranging from highly industrialized and urbanized centers to predominantly agricultural and rural provinces. Its nutritional profile also reflects the double burden of malnutrition, with both undernutrition and rising rates of overweight and obesity among children and adolescents (2). These considerations make Region IV-A a suitable and policy-relevant region for the present study.

The study included four schools from urban areas: Silang Central School in Cavite, Calamba Elementary School in Laguna province, and Cainta Elementary School and Rizal National Science High School in Rizal province; and two schools from rural communities: Tala National High School in Batangas province, and Carmona National High School in Cavite province. These schools were selected in coordination with the respective Offices of the Schools Division Superintendent and School Heads.

### 2.2 Sample and participants

A total of 178 students, both male and female, aged 10 to 18 years, participated in the survey component of the study. Students with chronic medical conditions lasting for 1 year or more, requiring ongoing medical care, or limiting activities of daily living were excluded. A target of 30 participants per school was set, yielding an intended sample of 180 adolescents across the six schools. Participants were identified by the School Heads or designated focal persons following the inclusion and exclusion criteria of the study. After applying the eligibility criteria, 178 students were included in the final sample.

### 2.3 Data collection

The school nutrition environment was assessed with a primary focus on the food environment, complemented by contextual data on the physical activity (PA) environment. Three methods were employed: a structured survey, geospatial mapping, and direct observation.

#### 2.3.1 Food environment

Data on the school food environment were collected using a pre-tested semi-structured survey, direct observation, and geospatial mapping. A survey among the students assessed the types and availability of foods and beverages within and around school premises and gathered information on their food purchasing behaviors and patterns. Through direct observation, the research team documented the types and number of food sources located within and outside the school premises. Photographs were taken to verify the observations.

External food sources surrounding each school were geolocated, and buffer zones of 50 m, 100 m, and 150 m radii were defined to assess the spatial distribution of food vendors. The distance of these food sources from each school was verified using Google Map. These spatial data were then used to generate food environment maps using QGIS Desktop 3.42.3 (19) software, providing a visual representation of the food landscape surrounding the schools.

#### 2.3.2 Physical activity environment

The school physical activity environment was assessed through direct observation, with focus on the availability, type, and condition of facilities that could support physical activity, such as playgrounds, open fields, sports courts, and gymnasiums. Observations were documented however, information on the frequency, duration, or actual use of these facilities by students were not collected.

### 2.4 Data analysis

Descriptive statistics were used to summarize the sociodemographic characteristics of the student respondents and their food purchasing behaviors and patterns. Spatial analysis was conducted to provide both a visual and quantitative representation of the school food environment, particularly focusing on the density and proximity of food sources relative to the school premises.

The density of food vendors within each buffer zone (0–50 m, 51–100 m, 101–150 m) was computed by visually counting the number of vendors, excluding the school canteen. The area of each concentric buffer zone (in m^2^) was calculated using the formula:

$A=\pi (R^{2}-r^{2})$ , where R^2^ is the inner radius and r^2^ is the outer radius. Vendor density per 1,000 m^2^ was calculated using the formula:


$$\text{Density}=(\frac{\text{Vendor count}}{\text{Area in}\;m^{2}})\times \;1000$$

### 2.5 Ethical considerations

Ethical approval for the study was obtained from the Food and Nutrition Research Institute Institutional Ethics Review Committee (FIERC-2024-012). Prior to participation, informed consent was obtained from parents or guardians, and written assent was secured from all student participants.

## 3 Results

**Figure 1:**
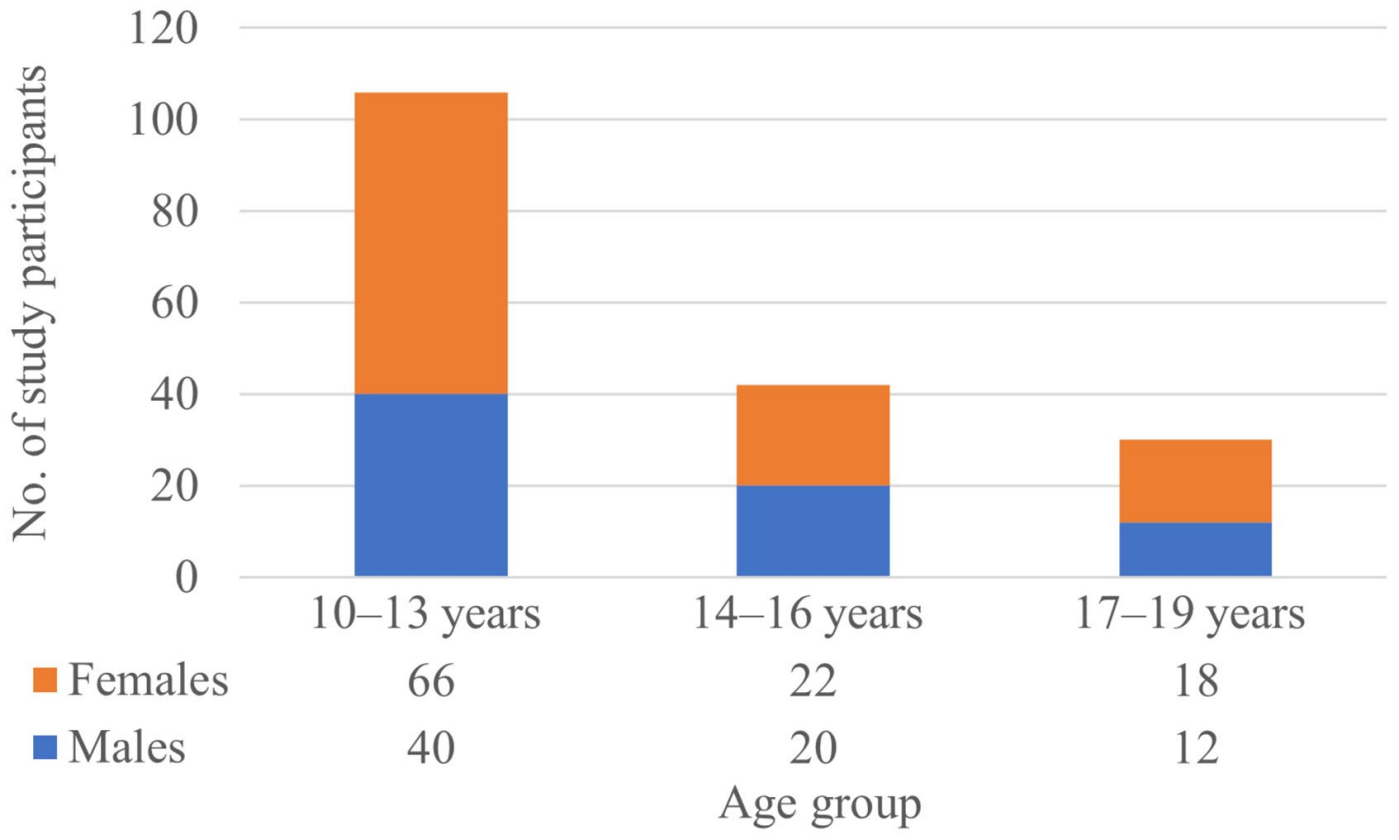
Distribution of study participants across age groups (early adolescence, middle adolescence, and late adolescence), by sex.

A total of 178 students participated in the study, with more females (*n* = 106) than males (*n* = 72). Early adolescence (10–13 years) comprised the largest age group for both sexes (Figure 1). The mean age was 13.39 years for males and 13.08 years for females.

### 3.1 Food environment

**Figure 2:**
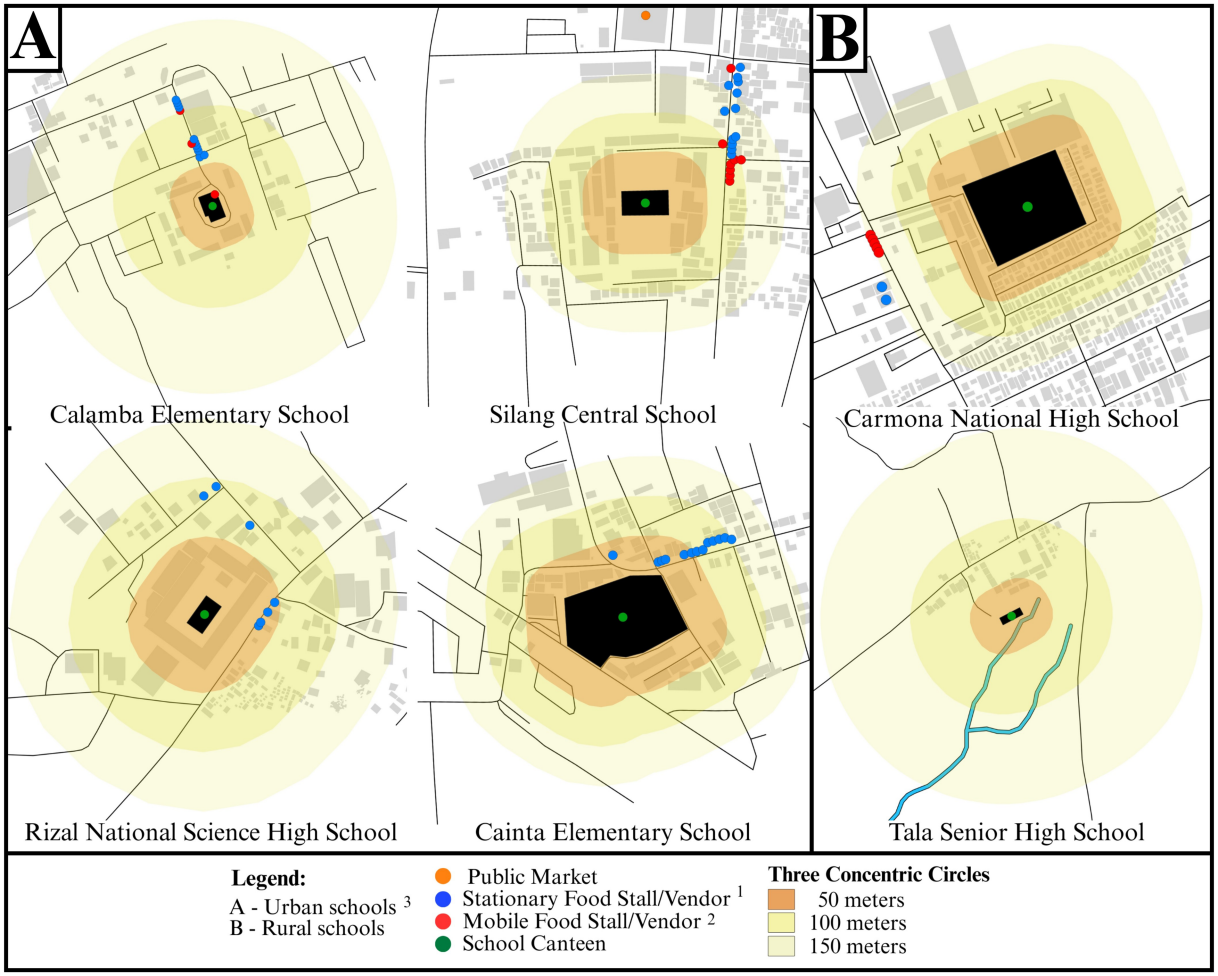
Spatial distribution of food vendors within a 150-meter radius of **(A)** urban schools and **(B)** rural schools. ^1^ Stationary Food Stall/Vendor includes restaurants, eateries, *carinderia,* convenience stores, *sari-sari stores,* snack and beverages stalls, *pasalubong stores/centers,* native delicacy shops, bakeries/bakeshops, milk tea/coffee shops. ^2^ Mobile Food Stall/Vendor includes food peddler, rolling snack and beverages stalls, rolling bakery. ^3^Urban-rural classification based on the Philippine Standard Geographic Code (PSGC) of the Philippine Statistics Authority (PSA) published 31 July 2025 (33).

The school food environment maps (Figure 2) revealed distinct spatial patterns of food source distribution within a 150-meter radius of each school. Across study sites, the school canteen was consistently situated within the innermost 50-meter zone, underscoring its proximity and central role in providing accessible food options to students. At Calamba Elementary School, a modest number of stationary and mobile vendors were found primarily along a single access route leading to the school. In contrast, Silang Central School has a more saturated food environment, with numerous mobile and stationary vendors concentrated along the perimeter road, particularly within the 50 to 100-meter buffer zone. The presence of a public market just beyond the 150-meter boundary further increased students’ exposure to diverse food options. Cainta Elementary School showed a high density of stationary food stalls, the majority located within the 100 to 150-meter buffer zone. Rizal National Science High School exhibited a moderate food environment, with vendors clustered mainly along adjacent roads within the 100-meter buffer zone. Carmona National High School demonstrated a more complex and denser vendor landscape. In contrast, Tala Senior High School lacked identifiable food vendors within 150-meters, likely due to its rural and isolated location characterized by fewer built structures and the presence of natural features such as streams. Consequently, students in Tala Senior High School relied more on the school canteen or home-prepared meals (“*baon*”).

Vendor density analysis supported these observations. The highest density was recorded in the 0–50 m of Silang Central School (0.89 vendors per 1,000 m^2^) (Table 1), indicating a high concentration of external food sources immediately adjacent to the school. Cainta Elementary School showed the highest vendor density in the 51–100 m range (0.30 vendors per 1,000 m^2^), while Calamba Elementary School and Carmona National High School exhibited a gradual decline in vendor density with increasing distance from the school. Rizal National Science High School showed a steeper decline in vendor density beyond the first 50 meters.

**Table 1:** Vendor density by distance zone across the study school sites (per 1,000 m^2^).

School	Distance zone	Area (m^2^)	Vendor count	Density (vendors/1,000 m^2^)
Calamba Elementary School	0–50 m	7,854	2	0.25
	51–100 m	23,562	3	0.13
	101–150 m	39,270	4	0.10
Silang Central School	0–50 m	7,854	7	0.89
	51–100 m	23,562	4	0.17
	101–150 m	39,270	1	0.03
Cainta Elementary School	0–50 m	7,854	3	0.38
	51–100 m	23,562	7	0.30
	101–150 m	39,270	2	0.05
Rizal National Science High School	0–50 m	7,854	3	0.38
	51–100 m	23,562	3	0.13
	101–150 m	39,270	1	0.03
Carmona National High School	0–50 m	7,854	2	0.25
	51–100 m	23,562	3	0.13
	101–150 m	39,270	0	0.00
Tala Senior High School	All zones	–	0	–

Table 2 summarizes the types of food and beverages available in and around the schools based on the student survey response (*n* = 178). Items were categorized into four main groups: cooked foods, packaged foods, fresh fruits, and beverages. Cooked foods included complete meals (e.g., rice with fried chicken, burger steak, *adobo*) and a variety of snacks (e.g., *siomai*, *turon*, fries, native delicacies). Packaged foods were widely available and subdivided into salty/savory snacks (e.g., chips, crackers, cup noodles) and sweet snacks (e.g., cookies, candies, cream-filled biscuits). Fresh fruits (e.g., apple, mango) were noted but comparatively limited. Beverages ranged from plain water and milk to sugar-sweetened beverages (SSBs) such as milk tea, sweetened juices, and soft drinks. As shown in Table 2, food and beverage types were largely similar across urban and rural schools, although some distinctions were observed. Urban schools offered a wider variety of commercially prepared foods and drinks such as *takoyaki*, *corndogs*, and *milk tea*, while rural schools featured more home-prepared and traditional items like *maruya*, *puto*, and *buko juice*. Across both settings, *fried snacks* and *sugar-sweetened beverages* were common.

**Table 2:** Types of food and beverages available in and around the school study sites in Region IV-A, Philippines.

Main category	Sub-category	Description	External food sources			School canteen		
			Urban schools	Rural schools	Both	Urban schools	Rural schools	Both
Cooked foods	Meals	Ready-to-eat dishes typically consisting of rice and a viand (e.g., meat, fish, vegetables)	*Liempo* with java rice, fried rice, hotdog, *ginataang gulay*, *tilapia*	*Siomai* rice	Fried chicken, burger steak	Fried chicken, *chao fan* rice, *tapa*, *pinakbet*, viand in coconut milk, fried rice, *sinigang*, *adobo*, tofu, pork steak, *longganisa*, ham, *tocino*, fried *tilapia*, *lomi*	*Giniling* rice, *menudo* rice, *pastil*, burger steak, *tinola*, pork dish, beef dish, *tocilog*, sausage,	Egg with rice, *sisig* rice, hotdog, *siomai* rice
	Snacks	Savory or sweet food items that are ready-to-eat but not considered full meals. These include finger foods, fried snacks, and baked items commonly consumed between meals.	Pancake, *lumpia*, corn, *corndog*, barbecue, *takoyaki*	Pizza, *turon*, *lumpiang gulay*, *isaw*, burger, *shanghai*, cheese sticks	*Siomai*, waffle, fries, *kikiam*, *kwek-kwek*, *fishball*, bread	*Champorado*, hotdog on stick, boiled banana, *kakanin*, *karioka*, *biko*, *bibingka*, barbecue, cheese sticks, boiled egg, brownies, doughnuts, hotdog bun, macaroons, *ginataang monggo*, steamed banana	*Lumpiang shanghai*, *maruya*, *lumpia toge*, *puto*, pasta dishes, kamote cue, banana cue, chicken balls, chocolate cake, hotdog bun, macaroons, corn, squid ball	*Lugaw*, spaghetti, *pancit*, *sopas*, *siomai*, *siopao*, pancake, pizza, burger, *turon*, waffle, *foot long*, sandwich, macaroni, *kwek-kwek*, *fishball*, bread, *ensaymada*, *mamon*, *kikiam*, fries
Packaged foods	Salty and/or savory	Packaged snack foods typically packaged and shelf-stable	Chips	Popcorn				Chips/curls, crackers, cup noodles, toasted bread, wafer, popcorn
	Sweet	Packaged treats with added sugars	Candies		Cookies, cream-filled biscuits	Coated cereals		Cream-filled/coated biscuits, sweetened curls/wafers/chips, cake bars, cookies, candies,
Frozen treats		Sweet, cold dessert-type items, typically dairy- or sugar-based, sold frozen and often consumed as snacks		Ice candy, snow cone, *mais con yelo*, ice banana	Ice cream, scramble	Ice cream, *mais con yelo*	Ice banana	Ice candy
Fresh fruits		Whole or sliced unprocessed fruits, typically sold raw without any additives or preparation beyond basic cleaning or peeling	Apple, mango, avocado					Mango
Beverages	Water	Plain drinking water, including bottled or filtered water, with no flavorings or additives		Water				Water
	Milk and alternatives	Dairy- or plant-based milk beverages (e.g., soy) sometimes fortified or sweetened			Milk	Milk, soy-based drinks		
	Sugar- sweetened beverages (SSBs)s	Beverages that contain added sugar, excluding milk-based drinks	*Palamig*		Milk tea, flavored shake/smoothie	Flavored water		Carbonated beverages, energy/ sports drinks, sweetened juice drinks, sweetened tea, *gulaman*, flavored shake/smoothie, *palamig*
	Fruit-based beverages	Juices made from fruits with no or minimal added sugar. These may be fresh-squeezed or bottled but not heavily processed or sweetened.		*Buko* juice		*Kalamansi* juice, avocado shake, *buko* juice, pineapple juice, cucumber juice	*Pandan* shake	Melon shake, mango shake
	Probiotic beverages	Cultured drinks designed to promote digestive health	Probiotic drinks					Probiotic drinks
	Other beverages	Drinks that do not fall under the above types	Coffee, tea					Chocolate drinks Coffee

**Figure 3:**
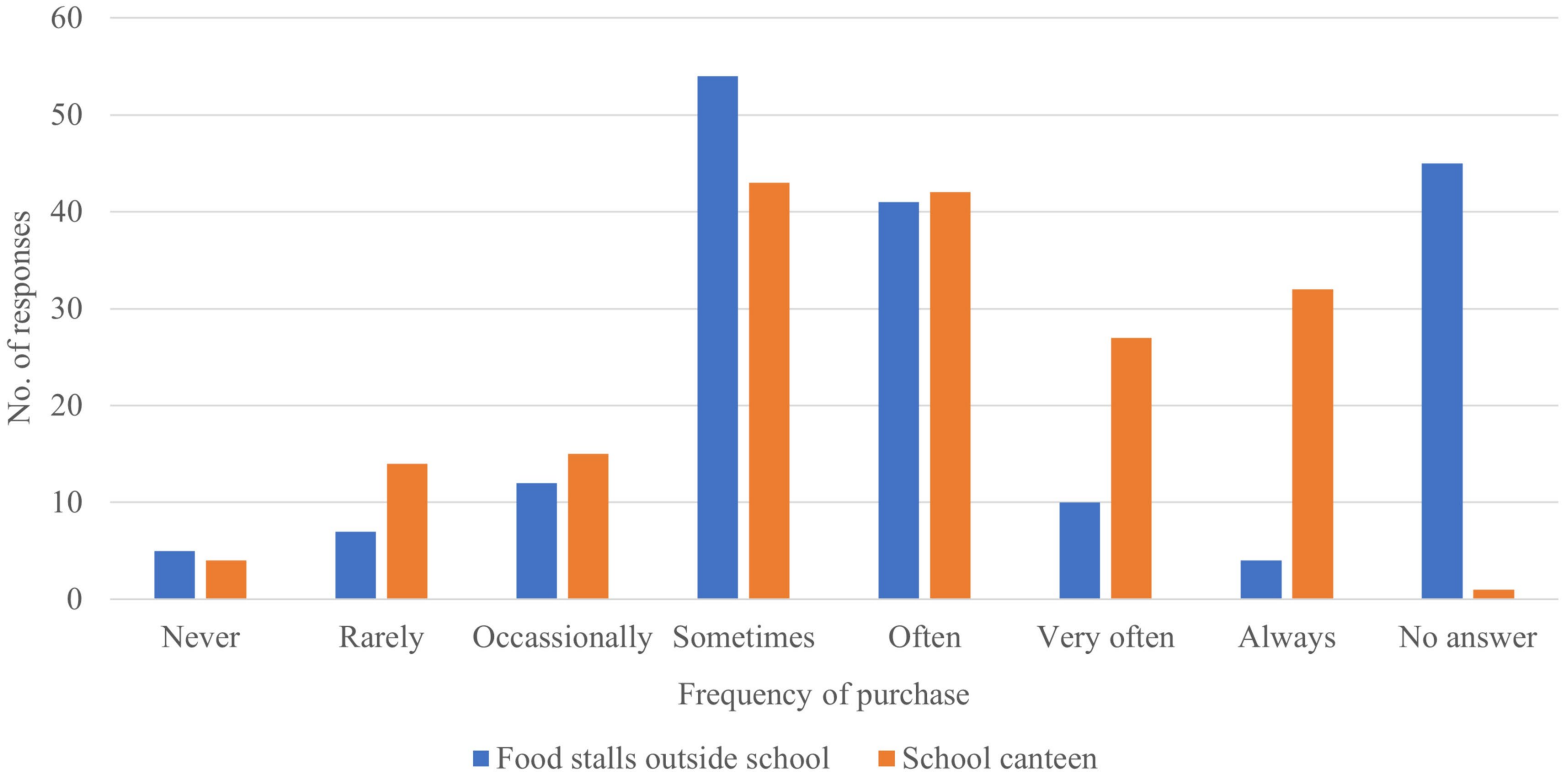
Frequency of food purchases from the school canteen and external food sources.

Student purchasing patterns (Figure 3) indicated that occasional purchases from food stalls outside the school (“sometimes” = 30%) were more common than from the school canteen (24%). However, frequent purchases (“often” = 24%; “very often” = 15%; “always” = 18%) were more often reported for the school canteen than with external food stalls (“often” = 23%, “very often” = 6%; “always” = 2%). A small proportion of students reported never purchasing from either food source (food stalls = 3%; school canteen = 2%). Notably, 25% of the respondents provided “no answer” for purchases from food stalls, compared to only 1% for the school canteen - suggesting potential underreporting or uncertainty about informal food purchases. Collectively, these findings imply that while students occasionally buy food outside school, their habitual food purchases remain anchored in the school canteen.

### 3.2 Physical activity environment

Spatial observation of school grounds revealed diverse layouts and infrastructure supporting physical activity in the six schools included in the study. Basketball courts were the most consistently available facilities, present in at least four schools, functioning as central, multifunctional spaces for both structured and informal student activities. Open grounds and green spaces were also common, offering flexible opportunities for active play. Covered walkways in Cainta Elementary School and Rizal National Science High School facilitated continuous pedestrian movement, contributing to the incidental physical activity among students.

Distinct spatial layouts influenced the level and type of physical activity opportunities across schools. Cainta Elementary School featured a compact concrete layout integrating school buildings, a feeding area, and a drop-off/parking zone, which may constrain movement patterns. On the other hand, Calamba Elementary School had a centrally located basketball court that enhanced visibility and accessibility. Carmona National High School showed a well-zoned, multi-nodal layout with multiple entry and exit points, and interconnected spaces conducive to varied movement patterns. Rizal National Science High School featured interconnected walkways forming the campus core, reflecting design principles that encourage internal navigation. Silang Central School is equipped with a spacious sports and/or recreation field, which facilitates a broad range of physical activities for students. Tala Senior High School had access to an oval track and field located outside the school perimeter, which suggests a shared-use or community-based facility. This could potentially extend opportunities to enhance physical activity among students.

## 4 Discussion

Research on the school nutrition environment in the Philippines remains limited, yet emerging evidence underscores its pivotal role in shaping children’s dietary behaviors and nutritional outcomes. The present study examined the food and physical activity environments of six public schools in Region IV-A, Philippines. Three key findings emerged. First, there were observable urban–rural disparities in the density and diversity of food sources surrounding schools. Second, school canteens were central to students’ daily food access across all study sites, although food offerings frequently mirrored those of external food sources, with limited availability of fruits, vegetables, and healthier beverages. Third, while all schools had some physical activity infrastructure, the quality, accessibility, and layout varied widely. Densely built campuses limited movement opportunities, whereas schools with larger grounds or shared community facilities showed greater potential to support active behaviors.

An important contribution of the present study is the generation of geospatial maps to quantitatively assess the proximity and density of food sources in and around the school study sites, which provides a nuanced understanding of the school food environment at the local setting. Geospatial mapping revealed marked variation in the distribution and density of food vendors within 150 meters of schools. Urban schools such as Silang Central and Cainta Elementary had high vendor density, especially within the 0–100-meter zone, whereas rural schools like Tala Senior High had almost none. This observed disparity extends prior work on the influence of geographic context and urbanicity on the food environment surrounding schools (20), highlighting the persistent and nuanced role of place in shaping food accessibility and availability. Prior studies have documented that food environments surrounding schools are not uniform across geographic areas, with urban and rural schools facing distinct but equally pressing challenges related to food access and availability (20–22). In urban settings, school food environments are often characterized by a high density and more diverse food sources, including fast-food retail outlets, convenience stores, and bakery and ice cream shops (20, 22). This saturation or “excessive” access to unhealthy food choices is conceptually referred to as “food swamp” (23). Conversely, rural school food environments are often marked by a relative lack or absence of food retail infrastructure, including both healthy and unhealthy outlets. While this may suggest a reduced risk of unhealthy food exposure, the lack of commercial activity in these areas can also create “food deserts” (23). In such cases, students may rely on limited food sources, like school canteens. These contextual disparities highlight the need for assessment frameworks that are sensitive to local conditions and can inform tailored, place-based interventions. Such frameworks should integrate geographic, socioeconomic, and cultural factors, as well as local policy environments that shape food retail patterns (20, 24).

In the present study, we noted that the school canteens are important food sources for students, particularly in areas with limited external vendors; however, the types of foods available in school canteens were often similar to those offered by external food sources, with both environments commonly providing energy-dense, nutrient-poor, ready-to-eat snack items such as SSBs, chips, deep-fried snacks, and processed baked goods Although the school canteens offer meal options, they are often served alongside processed meats, e.g., tocino, hotdog, and ham. In addition, items such as chips, candies, cookies, cream-filled biscuits, and flavored cereals dominate snack offerings in both vendor stalls and canteens. Perhaps most concerning is the minimal availability of fresh fruits, with only apples and mangoes occasionally observed in canteens, while external vendors do not appear to sell fresh fruits at all. Healthy fruit-based beverages with no added sugar are also rare or not prominent. These observations suggest fewer opportunities for healthier food choices and reinforce concerns about the quality of food environments within and around schools, where convenience and affordability may outweigh nutritional considerations in shaping students’ food purchasing behaviors.

Overall, our data reflect a school food environment marked by distinct urban–rural disparities, where the diversity of available food options, both within school canteens and from external vendors, is skewed heavily toward less healthy, energy-dense, and processed products. The apparent predominance of processed snacks, sugar-sweetened beverages (SSBs), and fried foods both inside and outside school premises reflects a persistent gap between policy intent and implementation. Despite the DepEd *Order No. 13, s. 2017* (“Policy and Guidelines on Healthy Food and Beverage Choices in Schools”), enforcement remains inconsistent. Challenges include unclear interpretation of the traffic-light system for classifying foods, limited resources for monitoring, and lack of delineated accountability mechanisms (13). Moreover, the policy does not regulate food sales immediately outside school premises, leaving a “proximity loophole” that exposes students to less healthy choices within meters of school gates.

At present, there is no national law in the Philippines specifying a minimum distance for food vendors from schools. However, local government ordinances in Quezon City and Pasig (25, 26) address this gap by restricting the sale and marketing of unhealthy foods within 100 meters of school perimeters. Both ordinances create task forces to oversee implementation and outline penalties for violations. Integrating these local regulations with national DepEd policies presents an opportunity for a multi-level, co-regulatory framework where national guidelines set the standards and LGUs operationalize enforcement. One key area for integration is geographic scope. The DepEd order covers only school premises and DepEd-sanctioned activities, while local ordinances regulate food sales and marketing within a 100-meter radius of school grounds. This spatial extension closes the “proximity loophole,” especially in urban settings where students often buy food just outside school gates. A second point of convergence is food classification. While the DepEd order uses the traffic-light system, local ordinances codify specific lists of allowed and prohibited food items. This enhances enforceability and reduces interpretative ambiguity for canteen operators and vendors. A third area involves governance and enforcement. Whereas the DepEd order relies on school administrators, local ordinances establish inter-agency task forces that include community stakeholders. This broader structure enables more effective monitoring and accountability while easing the administrative burden on schools. These integration points underscore the value of a co-regulatory model, i.e., national-level policy defines the standards, while LGUs enforce them through local ordinances, institutional mechanisms, and community engagement. In a decentralized governance system like the Philippines, such a multi-level approach supports stronger policy uptake and local adaptation.

In Region IV-A, local ordinances have been enacted to promote good nutrition and health among school children, reflecting increasing local government engagement in shaping healthier school food environments. For instance, Section 41 of City Ordinance No. 55–2015 s.2015 (*An ordinance enacting the health, sanitation, and safety code of the City of Bacoor and providing penalties for violations hereof*) in Bacoor City, Cavite mandates the display of nutrition information for cooked food items sold in food establishments, including canteens within both private and public schools. However, this provision notably excludes *carinderias* which are informal food sources that often provide affordable and convenient meals for students (27). This gap may reduce the ordinance’s effectiveness, as *carinderias* and other informal food outlets are common sources of food among students.

Similarly, the local government of Taytay, Rizal on the other hand, passed Ordinance No. 803, s.2024 or *An ordinance to protect children from the harmful impacts of food and beverage marketing*, which explicitly prohibits advertisements of food items high in fat, salt, or sugar, in child-centered settings like school zones, playgrounds, parks, and family mall areas (28). The ordinance also establishes a local task force chaired by the Municipal Mayor to oversee enforcement and monitoring. While this represents a progressive step toward reducing children’s exposure to unhealthy food marketing, implementation challenges and policy caveats remain. The ordinance primarily regulates advertising within defined geographic zones, leaving potential gaps through informal vendors, online platforms, and unregulated perimeters. Additionally, ambiguities in defining “high in fat, salt, or sugar” and the exclusion of small-scale or informal vendors may constrain the ordinance’s reach.

Despite these challenges, such local initiatives signal growing policy momentum in Region IV-A toward addressing concerns on school food environment. They offer valuable opportunities for policy learning and integration with national frameworks, particularly in operationalizing the DepEd’s Order No. 13, s.2017 on healthy food and beverage choices. Rapid urbanization in Region IV-A further underscores the need for localized approaches. With its blend of industrialized cities and agricultural municipalities, Region IV-A provides an ideal context for piloting interventions such as school-based pilot zoning policies, strengthened canteen monitoring, and partnerships with local vendors to promote healthier options. Regional DepEd offices, in collaboration with provincial health and nutrition councils, can serve as key intermediaries in scaling successful models across schools with similar urban–rural characteristics.

Findings in the present study further demonstrate substantial variability in the availability and spatial arrangement of physical activity (PA) facilities across schools. Basketball courts and open grounds were the most common, serving as both recreational and social spaces. However, limited space in dense urban schools such as Cainta Elementary constrained movement and informal activity. These observations support prior evidence that campus layout and accessibility of facilities influence students’ activity levels (29). Community-based or shared-use facilities, as seen with Tala Senior High School’s access to an external oval track, represent a promising strategy in resource-constrained settings (8, 30). Shared-use agreements can extend access to safe, structured spaces for physical activity beyond school hours and foster collaboration between schools, LGUs, and local sports programs.

Infrastructure alone, however, does not guarantee increased activity. The school social environment is equally important. Daily physical education, outdoor obstacle courses, extracurricular programs, and active encouragement to use sports facilities have all been linked to higher activity levels among adolescents (8). Effectiveness also depends on students’ attitudes toward Physical Education (PE). In the Philippines, engagement rises when PE includes enjoyable, interactive activities and positive teacher-student interactions (31). Beyond school grounds, active commuting, e.g., walking or cycling, offers another avenue for promoting physical activity. Supportive infrastructure such as bicycle lanes and pedestrian pathways can further encourage this behavior (32).

Together, these findings emphasize the need for multi-sectoral collaboration that integrates education, urban planning, and community development to create environments that holistically support youth physical activity.

### 4.1 Strengths, limitations, and future directions

This study provides an integrated assessment approach for school food and physical activity environments in selected public schools in Region IV-A, Philippines, combining spatial analysis, direct observation, and student survey data. A key strength lies in the use of geospatial mapping to objectively evaluate the density and proximity of external food vendors, alongside a structured inventory of school-based physical activity infrastructure. However, several limitations must be acknowledged. First, the purposive selection of study sites and participants may limit the generalizability of findings to other regions or school types, as this approach may have introduced some selection bias in both school and participant recruitment. Second, the cross-sectional design precludes causal inferences between environmental features and student behaviors. Third, the assessment of the physical activity environment was limited only to documenting the existing facilities within each school, but did not include an assessment of how these features influence students’ actual physical activity opportunities or behaviors. Future studies should combine environmental audits with behavioral or observational data to examine utilization patterns and their determinants. Additionally, self-reported data on food purchasing may be subject to recall bias or underreporting. Future research should consider longitudinal designs and incorporate objective measures of dietary intake and physical activity (e.g., wearable devices) to better capture behavioral outcomes. Expanding the study to include a more diverse set of schools, including private and geographically remote institutions, would further enhance the relevance of findings. Likewise, the conduct of qualitative research involving school staff, parents, and vendors would provide a more nuanced understanding of the barriers and facilitators to healthy school environments. Finally, a systems-oriented approach involving stakeholders from education, health, urban planning, and local governance is essential to translate these insights into actionable school and community-level policies.

## 5 Conclusion

This study highlights the crucial role of school nutrition environments in shaping the health and well-being of Filipino adolescents. While school canteens serve as central sources of food, the high density and proximity of external food vendors challenge efforts to promote healthy eating. Variations in school infrastructure and spatial layouts also influence opportunities for physical activity. These findings point to the need for comprehensive, context-sensitive strategies that integrate improvements in the food and physical activity environments within and around schools. Strengthening the national and local policy alignment, spatial and infrastructure planning, and intersectoral collaboration is vital to creating supportive school nutrition environments that encourage healthier behaviors among students.

## Data Availability

The original contributions presented in the study are included in the article/supplementary material, further inquiries can be directed to the corresponding author.
